# Inducible nitric oxide synthase, *Nos2*, does not mediate optic neuropathy and retinopathy in the DBA/2J glaucoma model

**DOI:** 10.1186/1471-2202-8-108

**Published:** 2007-12-19

**Authors:** Richard T Libby, Gareth R Howell, Iok-Hou Pang, Olga V Savinova, Adrienne K Mehalow, Joseph W Barter, Richard S Smith, Abbot F Clark, Simon WM John

**Affiliations:** 1 The Jackson Laboratory, Bar Harbor, ME, 04609, USA; 2 Alcon Research, Ltd., Ft. Worth, TX 76134, USA; 3 The Howard Hughes Medical Institute, Bar Harbor, ME, 04609, USA; 4 Department of Ophthalmology, Tufts University School of Medicine, Boston, MA, 02111, USA; 5 University of Rochester Eye Institute, University of Rochester Medical Center, 601 Elmwood Ave., Rochester, NY 14642, USA

## Abstract

**Background:**

Nitric oxide synthase 2 (NOS2) contributes to neural death in some settings, but its role in glaucoma remains controversial. NOS2 is implicated in retinal ganglion cell degeneration in a rat glaucoma model in which intraocular pressure (IOP) is experimentally elevated by blood vessel cauterization, but not in a rat glaucoma model where IOP was elevated by injection of hypertonic saline. To test the importance of NOS2 for an inherited glaucoma, in this study we both genetically and pharmacologically decreased NOS2 activity in the DBA/2J mouse glaucoma model.

**Methods:**

The expression of *Nos2 *in the optic nerve head was analyzed at both the RNA and protein levels at different stages of disease pathogenesis. To test the involvement of *Nos2 *in glaucomatous neurodegeneration, a null allele of *Nos2 *was backcrossed into DBA/2J mice and the incidence and severity of glaucoma was assessed in mice of each *Nos2 *genotype. Additionally, DBA/2J mice were treated with the NOS2 inhibitor aminoguanidine and the disease compared to untreated mice.

**Results:**

Optic nerve head *Nos2 *RNA levels varied and increased during moderate but decreased at early and severe stages of disease. Despite the presence of a few NOS2 positive cells in the optic nerve head, NOS2 protein was not substantially increased during the glaucoma. Genetic deficiency of *Nos2 *or aminoguanidine treatment did not alter the IOP profile of DBA/2J mice. Additionally, neither *Nos2 *deficiency nor aminoguanidine had any detectable affect on the glaucomatous optic nerve damage.

**Conclusion:**

Glaucomatous neurodegeneration in DBA/2J mice does not require NOS2 activity. Further experiments involving various models are needed to assess the general importance of *Nos2 *in glaucoma.

## Background

Glaucoma affects the vision of approximately 70 million people [1,2]. Optic nerve head excavation and degeneration of retinal ganglion cells (RGCs)are unifying characteristics of glaucoma. The causes of glaucoma are diverse and complex [3]. Many risk factors are known for glaucoma [3-5], with elevated intraocular pressure (IOP) being prominent [4-9]. IOP can be pharmacologically lowered, which in some cases can inhibit the development and progression of glaucoma [10-12]. However, in a significant number of cases, decreasing IOP does not prevent glaucoma progression. Identifying and targeting the mechanisms of RGC degeneration could help to protect the vision of more patients. Unfortunately, the molecular mechanisms of glaucoma are not well understood, and currently no therapies that target the neurodegeneration are commonly employed in clinical practice.

Nitric oxide has different roles in the central nervous system. Nitric oxide synthase 2 (*Nos2*; or inducible *Nos*, *iNos*) can be induced in response to a variety of external signals. In some settings, NOS2 activity plays an important role in inducing neurodegeneration [13,14]. Nitric oxide production, particularly through NOS2, has been implicated in ischemia-induced death in the retina [15-18]. The ability of a NOS2 inhibitor to protect RGCs from ischemia led to the suggestion that NOS2 inhibition may be protective in glaucoma [18]. The possibility that NOS2 inhibition could be neuroprotective in glaucoma was strengthened by a report showing that NOS2 inhibition delays RGC degeneration after axotomy [19].

Several lines of evidence point to a possible role for NOS2 in mediating glaucomatous neurodegeneration. In glaucoma patients, NOS2 expression was found to be up-regulated in optic nerve head glia [20-22]. The optic nerve head is believed to be an important site of damage in glaucoma [23-30]. In addition, human optic nerve head astrocytes can be induced to express NOS2 by either cytokines [22] or increased hydrostatic pressure [31]. NOS2 is induced in astrocytes of the optic nerve head in eyes of rats that have had intraocular pressure artificially increased by cauterization of the extraocular veins [32]. In the same rat model of glaucoma, pharmacological inhibition of NOS2 by aminoguanidine or L-*N*(6)-(1-iminoethyl)lysine 5-tetrazole amide reduced the number of RGCs that died [33,34].

The general importance of NOS2 in glaucoma was recently questioned by studies that do not support a role for NOS2 in another model of glaucoma [35,36]. Thus, the general importance of NOS2 induction for glaucoma is not certain. Considering these conflicting results from different induced rat models of glaucoma, it is important to further test the potential involvement of NOS2 in an inherited mouse model of glaucoma.

The DBA/2J mouse develops an inherited glaucoma with IOP elevation that is secondary to a pigment dispersing iris disease. DBA/2J glaucoma is similar to human glaucomas in that it is age-related and the pressure elevation is natural and variable [37-41]. The DBA/2 mouse has become one of the most widely used glaucoma models. The pressure elevation often leads to massive RGC loss, with the majority of eyes losing greater than 90% of their RGCs [40,42]. Reported differences in the timing and nature of neuron death in DBA/2 substrains point to a complex disease process that is affected by environmental as well as genetic differences between colonies of mice [40]. In our well characterized colony of DBA/2J mice, RGCs are specifically vulnerable to the glaucoma [28]. The pattern of RGC death supports involvement of axon injury in the optic nerve of DBA/2J mice [28,43] as well as in a closely related strain, DBA/2NNia [44]. In fact, detailed studies of the optic nerve support early injury of RGC axons as a key pathologic feature of DBA/2 glaucomas [45,46], with experimental evidence for an insult(s) damaging axons within the optic nerve head [45]. Considering the complex disease etiology, the specific vulnerability of RGCs, the characteristic pattern of RGC death and the early axon damage in the optic nerve head, DBA/2J glaucoma has important similarities to human glaucoma. For these reasons, we used DBA/2J mice in the current study. We used both genetic ablation and pharmacological inhibition of NOS2activity to test the involvement of NOS2 in glaucomatous damage to the optic nerve and retina in this inherited glaucoma model.

## Results

### *NOS2 *expression during DBA/2J glaucoma

**Figure 1:**
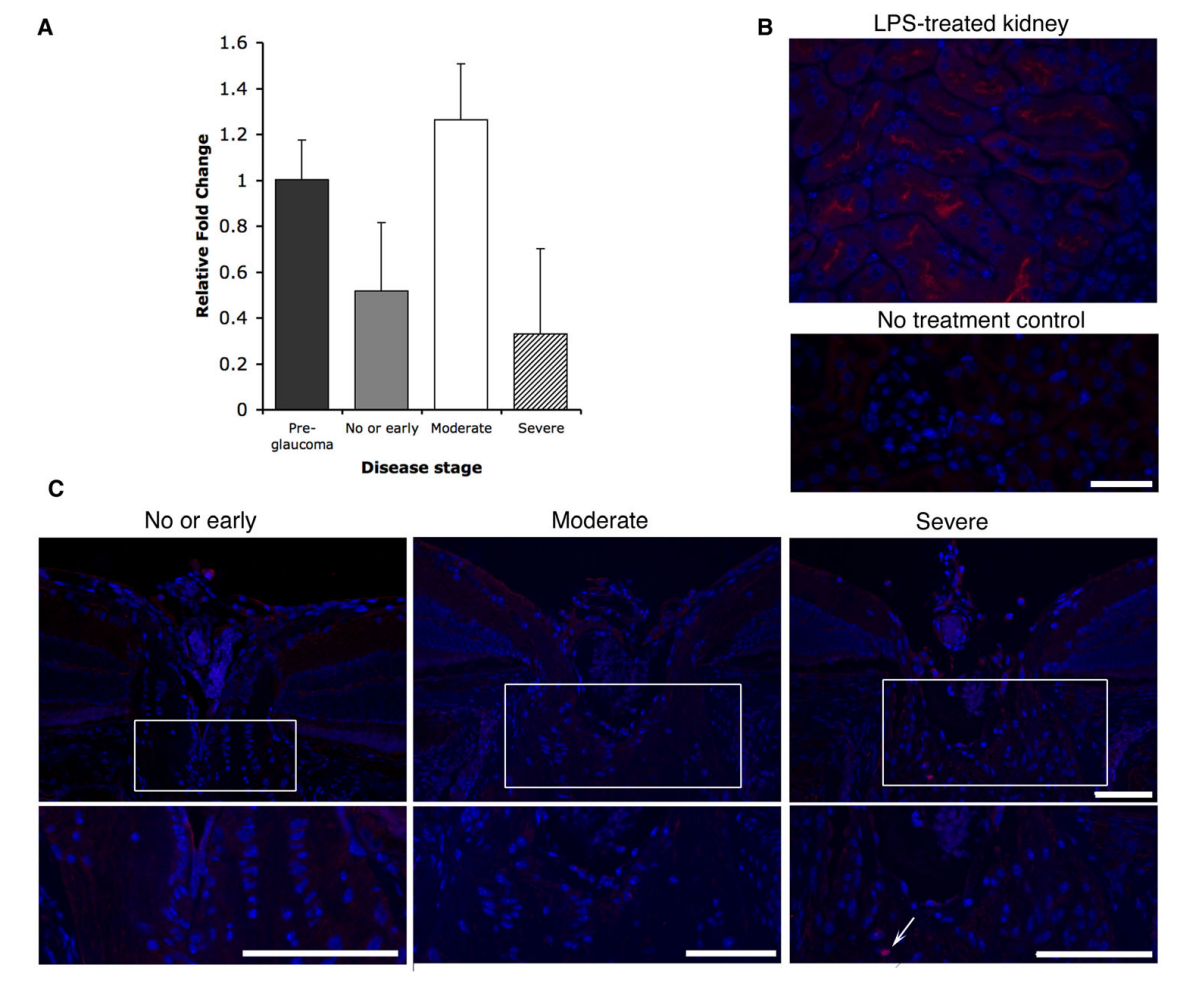
**NOS2 expression during glaucoma progression**. (**A**) Optic nerve head *Nos2 *RNA expression. Relative expression levels of *Nos2 *at different stages of glaucoma were determined using ΔCT ratios (see Methods). 'Control' eyes from 4 months old DBA/2J mice were considered to be pre-glaucomatous and showed no axon damage. 'Sample' eyes from 10.5 months old DBA/2J mice were assessed for glaucomatous axon damage and grouped into the no or early, moderate or severe stages of damage (see Methods). Values given are relative to pre-glaucoma controls. *Nos2 *expression was variable within each disease group (indicated by the SEM bars). Overall it decreased comparing pre-glaucoma with no or early glaucoma and increased modestly in eyes with moderate glaucoma (p < 0.05). (**B**) NOS2 protein localization in LPS-treated kidney (above) and no primary control (below). The NOS2 antibody shows strong and specific NOS2 staining (Red) in LPS-treated kidneys, with no non-specific binding of the secondary antibody. (**C**) NOS2 protein localization in the optic nerve heads of 10.5 months old DBA/2J mice, with the lower panels showing higher magnification of the boxed regions. There was no obvious correlation between NOS2 localization and glaucoma progression, although rare NOS2 positive cells were seen in eyes with severe glaucoma (arrow). All sections were processed, stained and imaged under identical conditions. The images shown here are representative of three different sections from three different eyes with the three different stages of glaucoma. Bars = 75 μm.

In some [19-22,32] but not all studies [35,36,47], increased expression of *Nos2 *is reported in glaucoma patients and rat models of glaucoma. To determine the temporal and spatial expression pattern of *Nos2 *in DBA/2J mice, we examined RNA and protein levels in the optic nerve head at different stages of glaucoma. The optic nerve head extending from the vitreal surface to approximately the non-myelinated-myelin junction of the optic nerve was dissected and used for RNA preparation. There was a modest increase of *Nos2 *RNA by quantitative real time PCR analysis in eyes with moderate axon damage, but *Nos2 *levels appeared to be lower in eyes with early or severe damage (Fig. 1A). Immunohistochemical analysis of sagittal sections did not detect NOS2 protein in either the retina or optic nerve head in the majority of eyes at each stage of disease. As a positive control, kidney sections from mice treated with lipopolysaccahride (LPS) were immunoreacted with the NOS2 antibody and NOS2 positive cells were detected (Fig. 1B). Rare cells that were NOS2 positive were identified in the optic nerve head of two severely affected eyes (Fig. 1C). Although these expression studies provided no clear association between NOS2 and glaucoma, NOS2 is a powerful modulator of cell functions that may be transiently expressed by single cells. Therefore, we continued to test the role of NOS2 in this glaucoma by both genetically and pharmacologically inhibiting its function.

### *NOS2 *deficiency does not alter the IOP profile of DBA/2J mice

**Figure 2:**
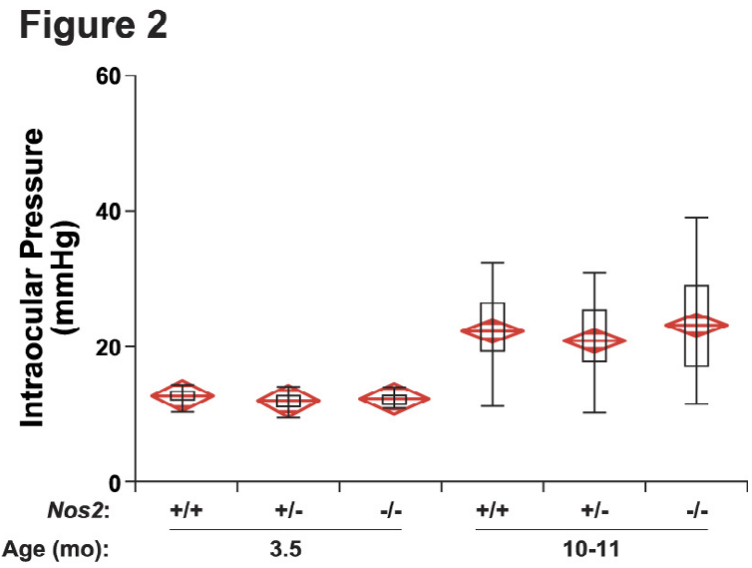
**NOS2 deficiency does not alter IOP**. The IOP distributions of mice of each genotype overlap extensively. The boxes show the upper and lower quartiles and the bars show the extremes including outliers. The centerline of each diamond is the mean with the upper and lower points of each diamond representing the 95% confidence intervals of the mean. Mean ± s.e.m in mm Hg, number of eyes examined; 3.5 months, *Nos*^+/+ ^12.6 ± 0.24, 18; *Nos*^+/- ^11.9 ± 0.30, 19; *Nos*^-/- ^12.2 ± 0.19, 19; P value for all comparisons > 0.08; 10–11 months, *Nos*^+/+ ^21.9 ± 0.87, 39; *Nos*^+/- ^21.0 ± 0.95, 32; *Nos*^-/- ^23.6 ± 1.3, 30, P value for all comparisons >0.09.

To investigate the involvement of *Nos2*, a null allele of *Nos2 *(*Nos2*^*tm*1*Lau *^[Nos2tm1Lau; [48]]) was backcrossed into the DBA/2J strain for greater than 13 generations. The genetic removal of *Nos2 *ensures that there is no enzymatic activity throughout life. Glaucoma in DBA/2J mice is the result of elevated IOP that is secondary to an iris disease. Preventing or lessening the iris disease alleviates IOP elevation and prevents the subsequent neurodegeneration [38,49,50]. Therefore, it is necessary to determine whether *Nos2 *genotype altered the iris disease or IOP profile. Slit lamp examination at various time points throughout the experiment showed that *Nos2 *genotype had no affect on the age of onset, progression, or severity of the iris disease (data not shown). Importantly, *Nos2 *genotype did not alter IOP in either young or aged mice (Fig. 2), and mice of all genotypes had a glaucomatous IOP elevation at 10–11 months of age (Fig. 2, P < 0.001, ANOVA, for all comparisons compared to young mice).

### *NOS2 *deficiency does not prevent glaucomatous neurodegeneration

**Figure 3:**
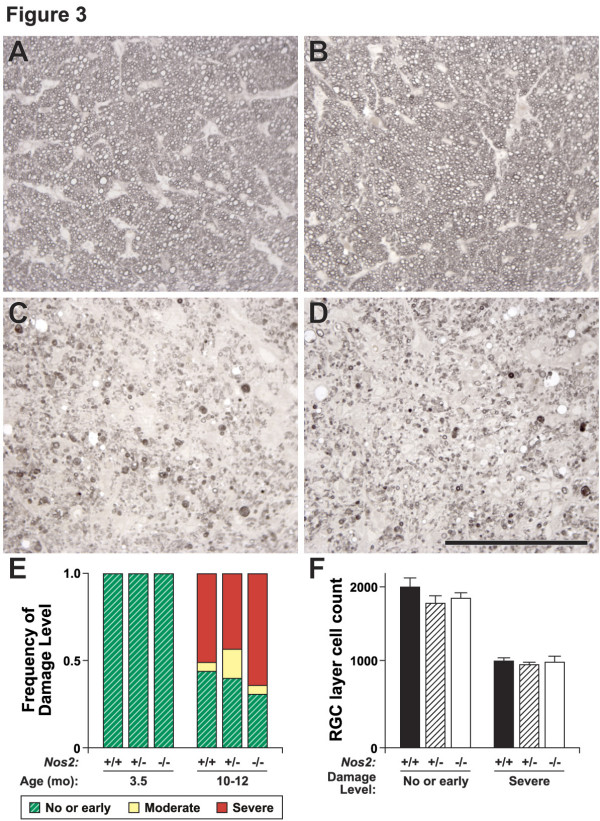
**NOS2 deficiency does not alter glaucomatous neurodegeneration**. (**A, B**) There were no gross anatomical differences between young *Nos2*^+/+ ^(**A**) and *Nos2*^-/- ^(**B**) nerves. (**C**) Eventually 80% of the optic nerves of DBA/2J mice develop severe optic nerve degeneration (*Nos2*^+/+^). (**D**) The complete absence of NOS2 did not prevent glaucomatous neurodegeneration as many *Nos2*^-/- ^nerves had severe axonal damage. (**E**) *Nos2 *genotype had no effect on the distribution of nerve damage. Numbers of nerves by age, genotype and disease state were: (given as total number analyzed, genotype, number of that genotype with mild, moderate, severe glaucoma) 3.5 months 6 *Nos2*^+/+ ^6,0,0; 7 *Nos2*^+/- ^7,0,0;11 *Nos2*^-/- ^11,0,0; 10–12 months 53 *Nos2*^+/+ ^23,3,27; 53 *Nos2*^+/- ^21,9,23; 56 *Nos2*^-/- ^17,3,36 (P value for all comparisons > 0.3). **F) **Average counts of RGC layer neurons from *Nos2 *mice of each genotype with either severe glaucomatous damage or no detectable glaucomatous damage (total number of cells of the 8 fields counted per retina; see methods). *Nos2 *genotype conferred no differential protective effect to RGCs in eyes with different degrees of axon loss. RGCs comprise 45–50% of cells in the RGC layer [71] and so almost all RGCs were lost in mice with severe axon loss independent of *Nos2 *genotype. Percentage of surviving cells; *Nos2*^+/+^, 54.4% ± 1.5; *Nos2*^+/-^, 54.6% ± 1.5; *Nos2*^-/-^, 58.0% ± 2.5; P values for all comparisons > 0.28. Number of retinal flat mounts counted, (given as genotype, mild, severe) *Nos2*^+/+ ^9, 7; *Nos2*^+/- ^12, 8; *Nos2*^-/- ^8, 13. Bar = 100 μm.

Some optic nerves from DBA/2J mice begin to show signs of glaucomatous neurodegeneration at 9 months of age [40]. The incidence and severity of glaucoma then increases rapidly over the next months, but not all mice develop glaucomatous nerve damage. Complete deficiency of NOS2 did not prevent homozygous mutant mice from developing severe glaucoma (Fig. 3C, D, E), showing that NOS2 is not required for glaucomatous axon degeneration in the optic nerve. Additionally, NOS2 deficiency did not lessen the amount of glaucomatous optic nerve degeneration in mice that were 10–12 months of age (Fig. 3E).

Although NOS2 deficiency did not affect axon degeneration, it is a formal possibility that it may affect apoptosis of the RGC somata. We have previously shown that BAX deficiency (BAX is a potent regulator of apoptosis) prevents somal apoptosis even when almost all RGC axons have degenerated in the optic nerve [42]. To assess if NOS2 deficiency protects RGC somata, even though it has no affect on their axons, we counted RGC layer cells for eyes whose nerves demonstrated different degrees of axon damage. *Nos2 *genotype had no affect on the number of RGC layer cells in mice with either 'no or early' or severe glaucomatous optic nerve damage (Fig. 3F). Thus, NOS2 deficiency does not differentially protect the RGC somata. Overall, our genetic studies detected no beneficial affect of completely removing NOS2 activity in glaucomatous DBA/2J mice.

### Aminoguanidine Treatment

The lack of detectable protective affect of genetically ablating NOS2 contrasts with the report of Neufeld and colleagues [51], who found that the NOS2 inhibitor aminoguanidine was neuroprotective in a rat model with induced IOP elevation. The outcome of these genetic and pharmacological studies may differ for a variety of reasons, including the possibility that the chemical inhibitor is not completely specific and has unknown off-target effects that may affect outcome. To better understand our findings in light of these reports, and to assess whether aminoguanidine has off-target effects that protect against glaucoma by a mechanism that is independent of NOS2, we tested the ability of aminoguanidine treatment to alleviate the glaucoma of DBA/2J mice.

Starting at either 3 or 5 months of age (2 different experiments), aminoguanidine (Ag) was administered in drinking water (1 mg/ml). Water consumption was measured for 50 Ag-treated and 50 non-treated mice, to determine the dose of Ag the mice received and whether Ag in the drinking water affected water consumption. Aminoguanidine did not affect the amount of water mice consumed (non-treated mice 4.0 ± 0.3 ml/day and Ag-treated 3.9 ± 0.2 ml/day; P = 0.736) nor did it affect their body weight even after being on the drug for 6 months (average g ± SEM, number of mice; control, 35.2 ± 1.0, 50; Ag-treated, 35.4 ± 0.9, 51; p = 0.89). This level of water consumption resulted in a calculated dose of 110 mg/kg/day. This dose is similar to that achieved by Neufeld and colleagues in their aged rats (mean rat body weight 0.575 kg, daily consumption 60 mg, dose 104 mg/kg/day).

### Aminoguanidine treatment does not alter IOP

**Figure 4:**
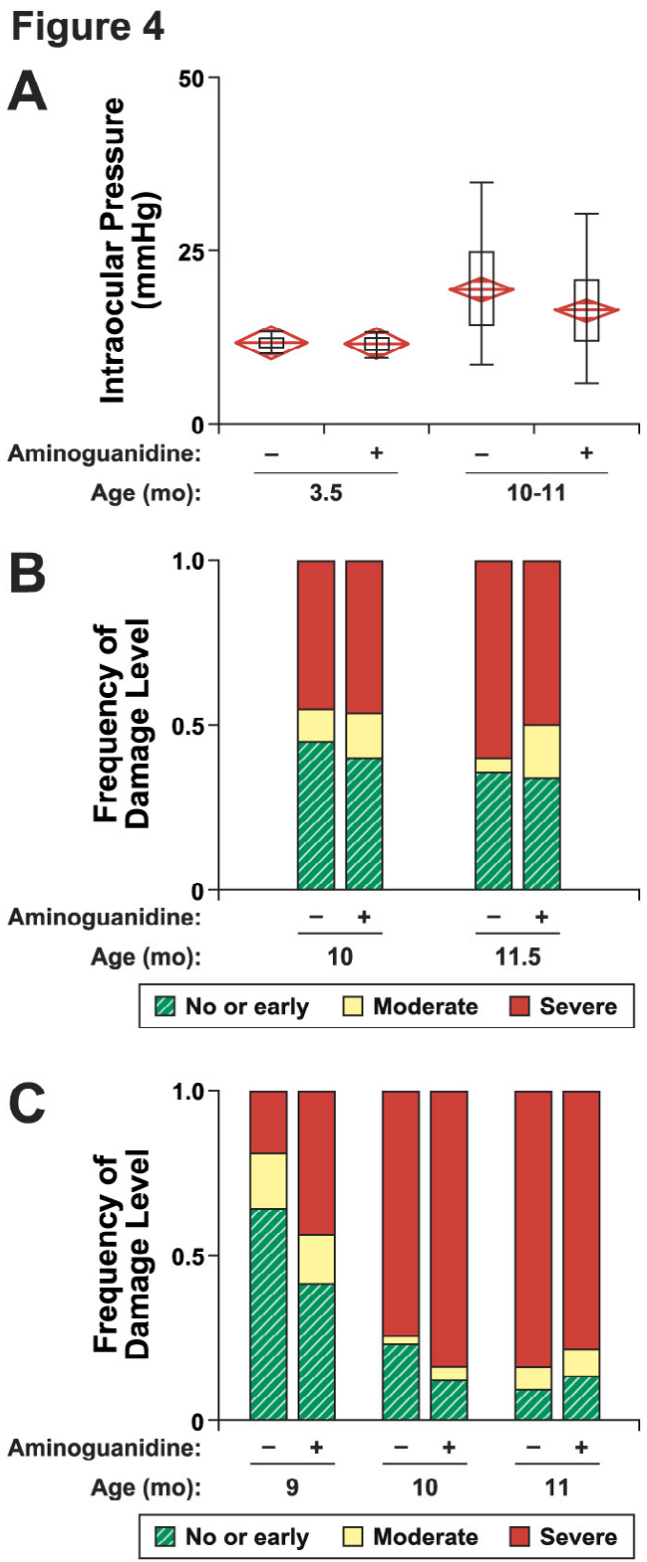
**Aminoguanidine treatment does not alter IOP or glaucomatous neurodegeneration**. **(A) **Ag-treatment did not affect the pre-glaucomatous IOP of DBA/2J mice (3.5 months: average mmHg ± s.e.m., number of eyes examined; control 11.8 ± 0.2, 19; Ag-treated 11.7 ± 0.2, 23; P = 0.76). Ag-treatment did not affect the ocular hypertension during the key window in disease progression, between 10 and 11 months (control, 18.2 ± 1.0, 31; Ag-treatment, 16.6 ± 1.3, 30; P = 0.334). (**B**) Ag-treatment from 5 months of age does not alter glaucomatous neurodegeneration (given as total number analyzed, treatment group, number of that treatment with mild, moderate, severe glaucoma) 10 months, 51 controls, 22,6,23; 51 Ag-treated 21,6,25; P = 0.293; 11.5 months, 55 controls, 18,6,31; 46 Ag-treated, 16,7,23, P = 0.896. (C) Ag-treatment beginning at 3 months of age does not protect from glaucomatous neurodegeneration. Nine months old treated mice actually tended to have more severe damage (given as total number analyzed, treatment group, number of that treatment with mild, moderate, severe glaucoma) 9 months, 42 controls, 27,7,8; 41 Ag-treated 17,6,18; P = 0.04; 10 months, 43 controls, 10,1,32; 49 Ag-treated, 6,2,41, P = 0.4; 11 months, 43 controls, 4,3,36; 37 Ag-treated, 5,3,29, P = 0.8. The experiments shown in B and C were performed at different institutions (see text).

Ag-treatment did not change the time course or the severity of the iris disease (data not shown). We first checked for any affect of Ag-treatment on IOP using young pre-glaucomatous DBA/2J mice. The IOP of young mice was not altered by Ag-treatment from 2 to 3.5 months of age. Similarly long-term Ag-treatment had no affect on IOP when assessed at 10 to 11 months, key ages of elevation in both sexes [39,40]. The IOPs of treated mice were significantly elevated compared to young mice (P < 0.001). Importantly, Ag-treatment did not alter the average IOP or the population distribution (Fig. 4A). The lack of effect of Ag-treatment on IOP is consistent with previous studies [35,51].

### Aminoguanidine treatment is not effective at preventing glaucomatous neurodegeneration

When treatment was started at 5 months of age using mice housed at The Jackson Laboratory, Ag-treatment did not alter the distribution of nerve damage at either 10 or 11.5 months (Fig. 4B; p > 0.2 for both ages). In fact, many mice developed severe glaucoma despite Ag-treatment. This lack of protection was confirmed with a second experiment using large cohorts of mice. These mice were housed and treated at Alcon Laboratories. The Ag treatment was the same except that it commenced at 3 months of age. For these mice, glaucomatous optic nerve damage was assessed at 9, 10 and 11 months of age (Fig. 4C). At no time point did Ag protect from degeneration, and at 9 months it tended to exacerbate the disease (P = 0.04). Interestingly, the damage was more severe in the second experiment. This fits with the variability of disease between different reports from different institutions and suggests that environmental factors alter disease progression in DBA/2J mice [40,44,52]. Overall, our combination of genetic and pharmacologic experiments provides no evidence for a role of NOS2 in this model of glaucoma.

## Discussion

Deciphering the mechanisms responsible for RGC dysfunction and demise in glaucoma is expected to lead to new neuroprotective treatments that will help to preserve the vision of more patients. NOS2 can be induced in nervous system microglia and astrocytes during disease, and NOS can act as an important mediator of neural cell damage and death [13,14]. NOS2 was reported to be induced in optic nerve glia during glaucoma, and involved in glaucomatous damage to the retina in a rat model of glaucoma [20-22,33,51]. Here we tested the involvement of NOS2 in glaucomatous neurodegeneration using the inherited DBA/2J model of glaucoma. There were modest changes in optic nerve head *Nos2 *RNA expression during the various stages of disease progression. Occasionally, NOS2 protein was detected in optic nerve cells with advanced glaucoma but not in unaffected optic nerves. However, neither the complete genetic ablation nor the treatment with an inhibitor of NOS2 had any affect on the severity or incidence of this glaucoma. Although our studies have not assessed if other nitric oxide synthases could have compensated for the absence of NOS2, as has been suggested in other systems [53], our results do not support a damaging role of NOS2 in this mouse model of glaucoma. We clearly demonstrate that glaucoma can manifest even when NOS2 is completely absent.

In contrast to our results, Neufeld and colleagues have proposed an important role for NOS2 in glaucoma and suggested its inhibition as a treatment [21,22,31-34,51,54-56]. These studies have provided a variety of data supporting a role for NOS2 in glaucomatous neurodegeneration. They have shown that NOS2 is expressed in the optic nerve in human glaucoma patients [20-22] and in a rat model of ocular hypertension [32]. NOS2 expression can be induced by increased hydrostatic pressure in cultured human astrocytes derived from human optic nerve heads [31]. Most intriguingly, using a rat model where IOP is artificially elevated by cauterization of extraocular veins, Neufeld and colleagues have shown that pharmacological inhibition of NOS2 could lessen glaucomatous RGC death [33,51].

Recently, Pang and colleagues [35] did not find evidence for a role of NOS2 in glaucomatous neurodegeneration. They induced elevated IOP in rats by injection of hypertonic saline into episcleral veins [57]. No significant increase in NOS2 expression (at the protein or message level) was found in optic nerve head cells due to ocular hypertension. Furthermore, they found no evidence for increased NOS2 in transected rat optic nerve heads or in optic nerve heads from glaucoma patients. Aminoguanidine treatment had no affect on glaucomatous damage in the rats. Also using episcleral injection of hypertonic saline in rats to induce elevated IOP, Kasmala and colleagues [36] found that another inhibitor of NOS2, L-*N*(6)-(1-iminoethyl)lysine 5-tetrazole (which has been shown to be protective in the vessel cauterization glaucoma model [33]) did not protect RGCs from death induced by elevated intraocular pressure.

There are multiple possible explanations for the differences in the observed role of NOS2 in glaucoma in these studies, including differences in genetically determined risk factors between the animals that were used. Pang and colleagues discuss many of the possibilities [35]. Here we limit discussion to a known difference between the studies of Neufeld and colleagues (which implicate NOS2) and other studies (which detected no effect of NOS2). Neufeld and colleagues cauterize extraocular veins to induce high IOP. This approach is more traumatic to vessels and surrounding tissue than the injection of hypertonic saline into extraocular veins (the method used by the other groups studying rats). The DBA/2J model is natural and does not involve experimentally-induced trauma to vessels. The greater vascular trauma in the cauterization model may somehow affect experimental outcome. It has been suggested that the extraocular vein cauterization method may induce retinal ischemia [58,59]. If true, ischemia could contribute significantly more to RGC death in the cauterization model than in the other models. NOS2 is clearly involved in ischemic retinopathy and NOS2 inhibitors (including aminoguanidine) can protect RGCs from ischemic injury in rats [16]. It will be important to assess ischemic markers in the various models to help determine if ischemia is more severe in the cauterization model and if this explains the differential efficacy of NOS2 inhibition.

Given the complexity of glaucoma, multiple different mechanisms are likely to contribute to disease in individual patients [3,58,60]. The relative importance of specific pathogenic mechanisms is likely to differ between individual patients and individual eyes due to differences in magnitude or duration of IOP elevation, environment, lifestyle or genetic constitution. Moreover, it is possible that different RGCs degenerate by different mechanisms even within an individual eye, depending on differences in protective or damaging factors in the local microenvironment. Mild or transient ischemia may contribute to RGC death in some patients [reviewed in [61-63]]. Thus, although we found no protective effect of NOS2 in the inherited DBA/2J model of glaucoma, it will be important to study the role of NOS2 in settings that have a greater ischemic component. Both IOP and blood pressure determine ocular perfusion pressure, and decreased ocular perfusion pressure may contribute to glaucomatous damage [64,65]. Thus, individuals with low blood pressure may be at greater risk of glaucomatous damage and their glaucoma may have a relatively strong ischemic component. Using DBA/2J mice, our future studies will assess these possibilities, including the potential protective role of NOS2 in a low blood pressure setting.

Genetic studies of human glaucoma have identified glaucoma genes and potential alleles that confer increased risk of disease. These genetic studies will help to define key pathologic mechanisms. One study reported a correlation between a regulatory region of the *NOS2 *gene and the development of primary open angle glaucoma [66]. However, in another study where the *NOS2 *gene was specifically assessed [67], NOS2 was not found to be associated with glaucoma. Studies of large patient populations are needed to exploit the full benefit of genetic studies for glaucoma. Continued studies utilizing a variety of animal models and human samples are clearly required to resolve the complex molecular pathways that determine RGC survival and death in glaucoma.

## Conclusion

NOS2 activity is not required for RGC death and associated axon degeneration in DBA/2J mice. Further studies are required to assess the general importance of *Nos2 *in glaucoma.

## Methods

### Animal Husbandry

All experiments were performed in compliance with the ARVO statement for use of animals in ophthalmic and vision research. All mice were bred at the Jackson Laboratory and maintained at The Jackson Laboratory or Alcon Laboratories. The mice were fed NIH31 (6% fat) chow ad libitum, and their water was acidified to pH 2.8 to 3.2. The environment was kept at 21°C with a 14 hour light: 10 hour dark cycle. The colony was monitored for specific pathogens by The Jackson Laboratory's routine surveillance program. A null allele of *Nos2*, *Nos2*^*tm*1*Lau *^[48], was backcrossed into DBA/2J for at least 13 generations. In the experiments using the null allele, only female DBA/2J mice were used. Furthermore, heterozygous by heterozygous matings were used so that mice of each genotype were littermates. Using this scheme protects from litter or cage-dependent differences as mice of each genotypes are housed together.

### Quantitative Real Time PCR

*Nos2 *RNA expression levels were assessed in 25 eyes from 10.5 months old DBA/2J mice (10 no or early, 6 moderate and 9 severe glaucoma – see optic nerve damage assessment) along with 10 'control' eyes from 4.5 months DBA/2J mice (pre-glaucoma). The front of each eye and lens were removed and the optic nerve head region dissected free using a sharpened 1 mm glass capillary. The ONH tissue was placed in RNAlater (Qiagen) for at least 24 hours then RNA was isolated using Microkit RNA extraction (Qiagen). For each eye, 35 ng of RNA was used to generate first strand cDNA using Message Sensor RT (Ambion). Expression levels for *Nos2 *(using primers GTCATGGCTTCACGGGTCAG and CCAGGTCCCTGGCTAGTGCT) and 3 control genes, *Gstm5 *(TCCTGCAGTCTGACCGCTTC and GCGAGCTCTGGCTCAGCATA), *Pias1 *(CATCTCTGCCAGCCACCAAC and TGGCCATGGCTCTCTCTCAG) and *Socs4 *(ACGTCCACTGCCTTGTTCCA and AGCAGAGCTTCGGCTGCATA) were determined for each eye. The 3 control genes were selected as they had the most consistent expression among 194 tested genes, irrespective of damage level (data not shown). Real time PCR reactions were prepared using Quant-iT Ribogreen RNA assay kit (Invitrogen) and run on the 7900 HT Fast Real Time PCR system (Applied Biosystems). For each eye, ΔCT values were calculated as CT(*Nos2*) – Geometric Mean of CTs for normalizers (*Gstm5*/*Pias1*/*Socs4*). The average of 'control' eyes was calculated and relative expression values for each of the 'sample' eyes was calculated as ΔCT (sample)/ΔCT (pre-glaucoma average). Finally, the average relative expression values and standard errors of the mean for each of the no or early, moderate and pre-glaucoma categories were calculated. Significance was assessed using Chi squared analysis (JMP 6.0.3)

### Immunohistochemistry

Whole eyes were immersion fixed in 4% paraformaldehyde in 1 × phosphate buffer for 24 hours at 4°C. Tissue was then either immediately processed and embedded in paraffin or was stored at 4°C in 0.4% paraformaldehyde in phosphate buffer. 8 μm paraffin sections were cut and processed for immunohistochemistry. NOS2 was visualized using rabbit anti-NOS2 (Santa Cruz, 1:200), with anti rabbit-Alexa 594 (Molecular Probes, 1;1000) as secondary. DAPI (Sigma) was used as a nuclear stain. Images were captured using Metamorph imaging software (Version 4.6, Universal Imaging, Downingtown, PA). All images were further processed with Adobe Photoshop and treated identically. As a positive control for NOS2, mice were treated with lipopolysaccahride (LPS), which is known to up-regulate NOS2 in the kidney. Eight hours after a single intraperitoneal injection of LPS (10 mg/kg) kidneys were harvested, processed and stained as described for eyes.

### Aminoguanidine Treatment

For mice in the aminoguanidine treatment group, 1 mg/ml of aminoguanidine was added to their drinking water. Treated and untreated water was changed on the same schedule (2 times a week). Treatment began at 2 months of age for the 3.5 months cohort and at 3 or 5 months of age for the 9–12 months old mice. Treated and untreated mice could not be in the same cage, but their cages were intermixed on the same shelves in the animal room and handled similarly. Roughly equal numbers of male and female mice were used for the 3.5 and 10–12 months old age groups. Significance was assessed using Chi squared analysis (JMP 6.0.3)

### Iris Disease and Intraocular Pressure Measurement

The experimenter was unaware of the genotype or treatment group of the mouse at time of the examination. A slit-lamp bio-microscope was used to track the iris disease that is present in DBA/2J mice as previously reported [37,39,40,68]. The microneedle method of IOP measurement was used as previously described [69-71]. Ages and sample sizes are detailed in the results. Statistical analysis was by ANOVA.

### Optic Nerve Damage Assessment

Retro-orbital optic nerves were fixed, processed, embedded in plastic, and sectioned as previously reported [40,42,72]. Sections were stained with paraphenylenediamine (PPD), which stains all myelin sheaths, but differentially stains the axoplasm of sick or dying axons darkly. This method is more sensitive at detecting glaucomatous damage than axon counting. This is because individual damaged axons can be detected by PPD staining while substantial axon loss is necessary to detect differences when comparing axon counts (due to the considerable variability in axon number from eye to eye). The 362 optic nerves used in this study were independently determined to have either no or early, moderate, or severe glaucomatous damage by multiple masked investigators (see below). Axon counts on randomly selected nerves with each damage level have demonstrated that each assigned level represents a clearly separable stage of disease [42,73]. To assign a damage level, the investigators analyzed each nerve for the relative number of healthy and damaged axons as well as the amount of gliosis. In nerves with no or early glaucoma fewer than 2% of the axons are lost or damaged. This mild level of damage is routinely observed in aged-matched non-glaucomatous mice and so we have previously called this damage level mild but not considered it a definitive stage of glaucoma. Some of these eyes must be at early stages of glaucoma that are not detectable by these methods while others have no glaucoma. Thus, we have reclassified this damage level as no or early glaucoma when studying aged mice. Nerves with no or early glaucoma have 51195 ± 934 (mean ± SEM, n = 18) axons and are not statistically different from young pre-glaucomatous DBA/2J nerves 52074 ± 451 (p > 0.05) (n = 10). Moderately affected nerves have numerous sick and degenerating axons throughout the nerve, but the majority of axons appear healthy. Nerves with this level of damage have typically lost approximately 30% of their axons and on average have 31410 ± 2199 (n = 8) axons remaining. Severe nerves have lost more than 50% of their axons, and many severe nerves have lost the vast majority of their axons(5454 ± 1211, n = 24). Of the 390 nerves used in this study, the first two investigators assigned the same damage level 87% of the time. If the two investigators did not assign the same damage level, a third masked investigator graded the nerve. The first two investigators never differed by more than one damage level and the damage level determined by the third investigators always matched that of one of the first two investigators. The final damage level was assigned by consensus (the most common damage level assigned). The nerve damage distributions of the different genotypes or treatment groups were compared by Chi square analysis (JMP 6.0.3).

### Retinal Flat Mounts

Retinal flat mounts were prepared and RGC layer cells counted as previously described [42]. Briefly, eyes were removed and fixed overnight at 4°C in 4% paraformaldehyde in 1 × phosphate buffer. After fixation eyes were rinsed, processed and flat-mounted onto a microscope slide with the RGC layer face up. The RGC layer was stained with a modified Nissl stain. Eight 40× fields, consisting of two fields from the periphery of each retinal quadrant were electronically photographed and counted (manually) using Metamorph software. The sum of all eight fields was used for the total for each retina. Age matched retinas with corresponding optic nerves that had no signs of glaucomatous damage were used as controls for the glaucomatous retinas. Significance (P < 0.05) was determined using the student's t test.

## Authors' contributions

SWMJ and AFC conceived the study and oversaw all components including manuscript preparation. RTL, GRH, IHP, SWMJ and AFC participated in the design of the experiments and the analysis of data. RTL, GRH and SWMJ wrote the manuscript. SWMJ and IHP treated the mice with aminoguanidine and OVS participated in IOP and tissue collection. AM performed real time PCR and participated in analysis of data. JB performed RGC layer counts and participated in analysis of data. RSS performed clinical analyses and participated in assessing optic nerve damage levels. All authors read and approved the final manuscript.
